# Whites Improved Stomach Pump, and Cupping Apparatus

**Published:** 1834

**Authors:** 


### VI. White's improved Stomach Pump and Cupping Apparatus.

We have lately examined a small box containing a pump, to which is attached a tube for emptying the stomach, another for administering enemata, and another for injecting the colon; also, nipple glasses, and cups for drawing blood. With all these valuable facilities, the apparatus is quite portable, and sells at a price to bring it within the means of every physician. It is peculiarly adapted to the wants of our brethren in the country.

The proprietor for the western states, Dr. L. B. Cotes, has obtained certificates in its favour from the entire medical faculty of Transylvania University, and two of the professors in the Medical College of Ohio, all of which are expressed in terms of decided approbation. We concur with these gentlemen in all they have said.

